# Conditional disease-free survival among patients with breast cancer

**DOI:** 10.1097/MD.0000000000005746

**Published:** 2017-01-10

**Authors:** Hyun-June Paik, Se Kyung Lee, Jai Min Ryu, Sungmin Park, Isaac Kim, Soo Youn Bae, Jonghan Yu, Jeong Eon Lee, Seok Won Kim, Seok Jin Nam

**Affiliations:** a Division of Breast and Endocrine Surgery, Department of Surgery, Samsung Medical Center; b Department of Surgery, Samsung Medical Center, Samsung Biomedical Research Institute, Sungkyungkwan University School of Medicine, Seoul, Korea.

**Keywords:** breast neoplasms, conditional survival, disease-free survival, hormone receptor

## Abstract

Conditional disease-free survival (CDFS) reflects changes over time. Because traditional disease-free survival (DFS) is estimated from the date of diagnosis, it is limited in the ability to predict risk of recurrence in patients who have been disease free. In this study, we determined CDFS of breast cancer patients and estimated the prognostic factors for DFS.

We retrospectively reviewed clinical data of 7587 consecutive patients who underwent curative surgery for breast cancer between January 2004 and December 2013 at Samsung Medical Center. Univariate and multivariate analyses were performed to identify risk factors for DFS, which was computed using the Kaplan–Meier method. CDFS rates were based on cumulative DFS estimates.

Median follow-up duration was 20.59 months. Three-year DFS was 93.46% at baseline. Three-year CDFS survival estimates for patients who had been disease free for 1, 2, 3, 4, and 5 years after treatment were calculated as 92.84%, 92.37%, 93.03%, 89.41%, and 79.64%, respectively. Three-year CDFS increased continuously each year after 1 year of DFS in hormone receptor (HR)-negative patients but decreased each year in HR-positive patients.

In HR-positive patients who are disease free after 3 years, continuous care including surveillance and metastases workup should be considered, although this is not recommended in the current guidelines. On the other hand, the social costs may be reduced in HR-negative patients by extending the surveillance interval. Further studies are needed to identify indicators of DFS prognosis in breast cancer patients.

## 1 Introduction

Breast cancer is the most common cancer among women worldwide, with an estimated 1.67 million new cases diagnosed in 2012. This represents about 25% of all cancers and 12% of all new cancer cases in women. Breast cancer is the most common cause of cancer death in women in less developed countries (14.3%) and the second most common cause of cancer death in more developed countries (15.4%). Because of the favorable survival of breast cancer patients, the mortality rate is less than the incidence.^[1]^ The methods traditionally used to evaluate survival are based on measures made at the time of diagnosis. Although these indicators provide much information to patients and physicians, they have less value for those patients who survive for a considerable time after the diagnosis. The term conditional disease-free survival (CDFS) seems to provide a better indicator of the risk over time and may give more accurate information for patients who survive for a certain period of time.^[2–4]^

Breast cancer is a heterogeneous disease whose diagnosis, prognosis, and treatment depend on the molecular subtype rather than a single characteristic.^[5]^ Therefore, a tailored follow-up treatment is determined according to the factors that significantly affect the DFS of breast cancer patients. Understanding more about CDFS after diagnosis and treatment in breast cancer patients may help the clinician develop the plan for the follow-up period. For example, certain patients may need more intensive management during the follow-up period, whereas other patients may not need routine checks or the intervals between examinations can be lengthened. In this study, we analyzed CDFS according to traditional prognostic factors such as molecular subtype and pathological stage,^[6,7]^ and we tried to identify indicators that could predict recurrence in patients.

## 2 Methods

### 2.1 Patients

Approval was granted by the Institutional Review Board of Samsung Medical Center (IRB file No. 2015-07-142). To protect the personal information, patient records and information were anonymized and deidentified before analysis. We retrospectively reviewed the clinicopathological records of 8938 patients diagnosed with invasive breast cancer who received surgical treatment at Samsung Medical Center between January 2004 and December 2013. Patients with incomplete data were excluded, and 7587 cases remained and were analyzed. The demographic and clinicopathological data abstracted from medical records included age, body mass index, tumor site, date of surgery, date of recurrence, breast surgery type (total mastectomy or breast-conserving surgery), axillary surgery type (sentinel lymph node biopsy or axillary lymph node dissection), histopathology, nuclear grade, pathological stage, and immunohistochemical status (Table 1). Recurrence was defined as the return of disease, proven by biopsy, after surgical treatment or a suspicious lesion regarded as distant metastasis on circumstantial evidence. DFS was defined as the period after surgery without recurrence.

**Table 1:**
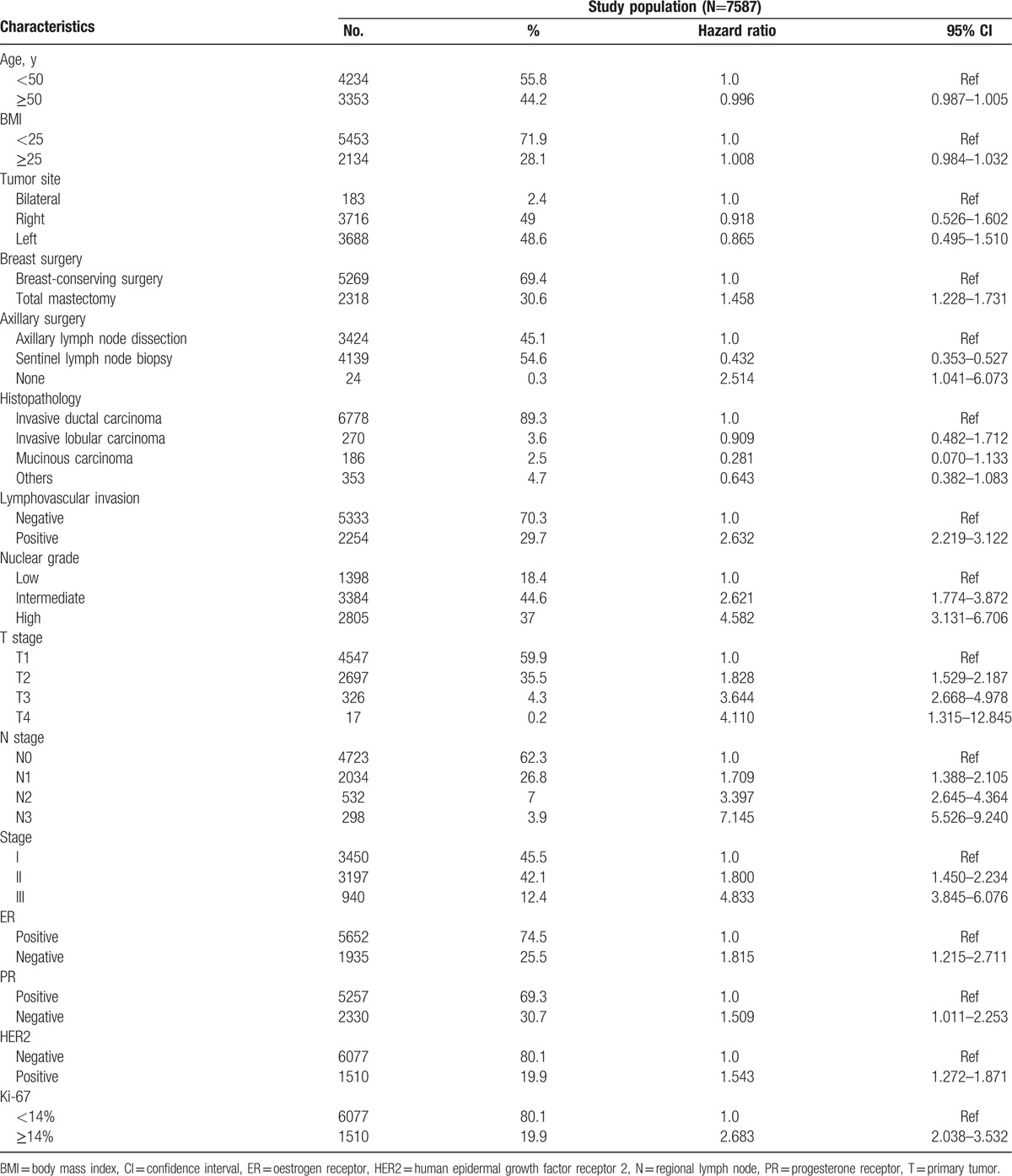
Demographic and clinicopathological characteristics of the study population with hazard ratios and corresponding 95% CIs for recurrence at baseline.

### 2.2 Immunohistochemistry and breast cancer subtype

A positive estrogen receptor (ER) or progesterone receptor (PgR) test is defined as positive staining of greater than or equal to 1% of tumor cells. And a negative test is defined as staining of less than 1% of tumor cells. In case of either ER or PgR is positive, hormone receptor status is positive. If both receptors are negative, hormone receptor status is negative.

HER2 status was evaluated according to the 2013 American Society of Clinical Oncology (ASCO)/College of American Pathologists.^[8]^ Circumferential membrane staining that is complete, intense, and within >10% of tumor cells results in a score of “3+.” Circumferential membrane staining that is incomplete and/or weak/moderate and within >10% of tumor cells or complete and circumferential membrane staining that is intense, and within ≥10% of tumor cells is scored “2+.” Incomplete membrane staining that is faint/barely perceptible and within >10% of tumor cells is “1+." No staining is observed or membrane staining that is incomplete and is faint/barely perceptible and within ≥10% of tumor cells is “0.” A positive test is defined as staining of 3+ score. The score of 2+ is interpreted as equivocal. And a negative test is defined as staining of 0/1+ scores.

Breast cancer subtypes are defined according to the recommendations of the 13th St Gallen International Breast Cancer Conference.^[9]^ The definition of luminal A subtype we used is ER and PgR positive, HER2 negative and Ki-67 <14%. Luminal B subtype is defined as ER positive, HER2 negative and Ki-67 ≥14% or ER positive and HER2 positive. Subtypes described in this study are all based on tissues at the date of diagnosis or surgery.

### 2.3 Statistical analysis

All data were analyzed by using SAS version 9.4 (SAS Institute Inc, Cary, NC) and all graphs were made using R 3.2.2 (Vienna, Austria; http://www.r-project.org/). DFS estimates were calculated using the Kaplan–Meier method with the date of surgery as the time origin. The effects of relevant patient characteristics on DFS were assessed using Cox proportional-hazards models. The hazard ratios were estimated with 95% confidence intervals (CIs). Differences were considered significant at *P* values <0.05.

Univariate CDFS analysis of risk factors for breast cancer recurrence was performed at 0, 1, 2, 3, 4, and 5 years after the operation. All risk factors selected at least once in each univariate analysis were used together in the multivariate CDFS analysis at 0, 1, 2, 3, 4, and 5 years after the operation (Table 2).

**Table 2:**
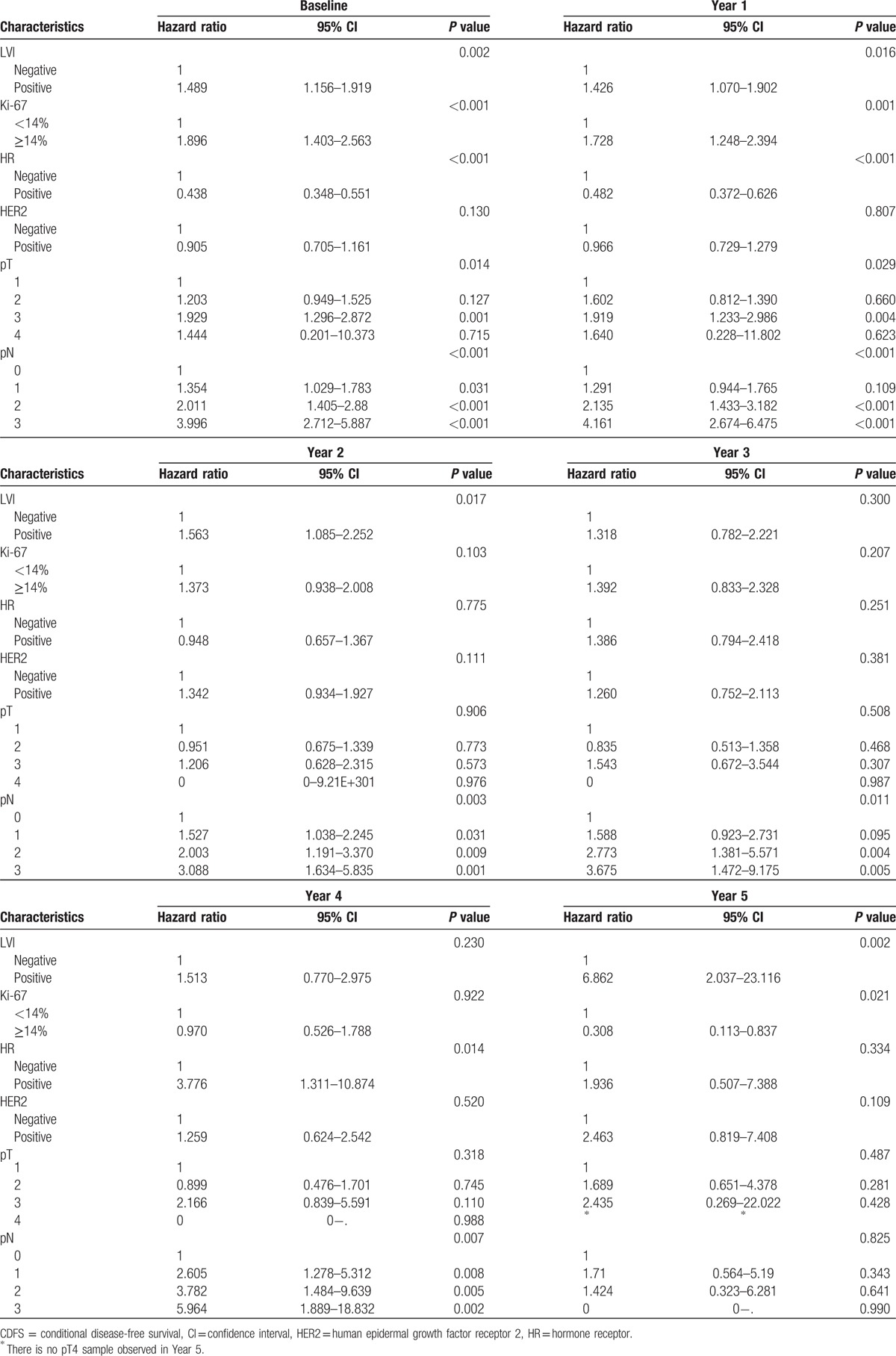
Multivariate CDFS analysis of risk factors for disease-free survival after breast cancer surgery.

CDFS is defined as the probability of surviving a further y years given that a patient has already survived x years after the diagnosis.^[2]^ CDFS was calculated as the probability of remaining disease free for an additional y years (CDFS_y_) given that a patient has survived for x years. We set y to be 3 in our study and used cumulative DFS (CuDFS) estimates to compute CDFS estimates. For instance, the 3-year CDFS estimate for patients who had already been disease free for 1 year was computed by dividing the 4-year CuDFS by the 1-year CuDFS, which is summarized as CDFS_3_ = CuDFS_(x+3)_/CuDFS_(x)_.

## 3 Results

The demographic and clinicopathological characteristics of the 7587 patients included in our study are summarized in Table 1. The median age of our study population was 49.2 years. Ninety percent of the patients were diagnosed with invasive ductal carcinoma and 46% of the patients were in stage I. Seventy-five percent of the patients were ER positive, whereas 70% of the patients were PgR positive.

**Figure 1:**
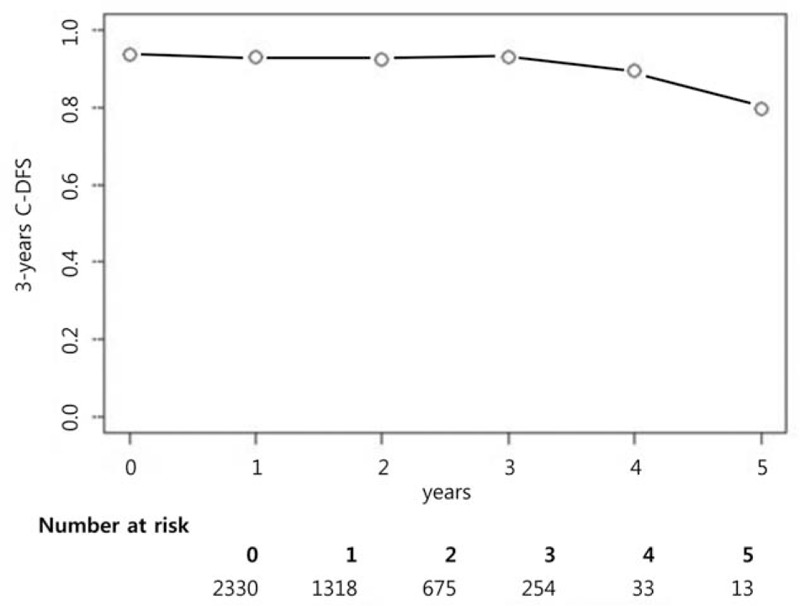
Three-year conditional disease-free survival (CDFS) estimates in breast cancer patients.

Three hundred fifty-three (4.65%) patients were diagnosed with recurrent breast cancer and median follow-up duration calculated by reverse Kaplan–Meier estimator was 20.59 months (95% CI, 19.47–21.61 months).^[10,11]^ At baseline, the 3-year DFS was 93.46%. The 3-year CDFS survival estimates for patients who had been disease free for 1, 2, 3, 4, and 5 years after treatment were calculated as 92.84%, 92.37%, 93.03%, 89.41%, and 79.64%, respectively (Fig. 1).

At year 0 (baseline), positive lymphovascular invasion (LVI), Ki-67 labelling index ≥14%, high pathologic primary tumor (pT) stage, and high pathologic regional lymph node (pN) stage were risk factors. On the other hand, positive hormone receptor (HR) status was a preventive factor. Risk factors and preventive factors at year 1, year 2, year 3, year 4, and year 5 changed differently as time passed (Table 2).

**Figure 2:**
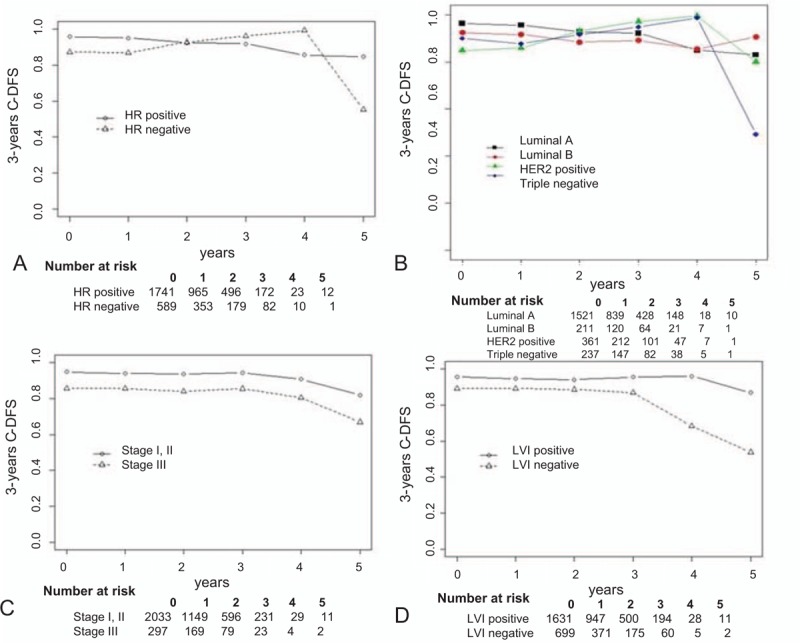
Conditional disease-free survival (CDFS) stratified by (A) hormone receptor (HR) status, (B) molecular subtype, (C) pathological stage, and (D) lymphovascular invasion (LVI).

Figure 2 shows the results of 3-year CDFS stratified by HR status, molecular subtype, pathological stage, and lymphovascular invasion status. In the HR-negative group, after 1 year of DFS, 3-year CDFS increased continuously each year. By contrast, in the HR-positive group, 3-year CDFS decreased continuously each year. Until 2 years of DFS, CDFS was higher in the HR-positive group, but this trend was reversed after 2 years of DFS, when CDFS became higher in the HR-negative group. A similar result was obtained when the groups were stratified by molecular subtype. The 3-year CDFS of luminal A and luminal B subtype patients decreased continuously, whereas the 3-year CDFS tended to increase continuously increase before year 4 and decrease at year 5 in patients with the HER2 subtype or triple-negative subtype. By contrast, 3-year CDFS stratified by pathological stage and lymphovascular invasion status showed similar trends in the groups.

## 4 Discussion

Traditionally, the accepted indicators for predicting the recurrence of breast cancer after treatment are molecular subtype, pathological stage, and age.^[6,7,12,13]^ However, studies that have used these indicators for breast cancer patients were based on the DFS, which is obtained using parameters measured at the time of diagnosis. Survival rates estimated from the time of diagnosis have less meaning for patients who have survived for a certain period after diagnosis.^[2,3,14]^

Therefore, the conditional survival rate has been introduced to predict survival in patients with various cancers who have already survived a period of time after the diagnosis.^[4,15–18]^ The concept of conditional survival was first introduced in 1979.^[19]^ Henson and Ries^[2]^ showed the importance of conditional survival to breast cancer patients in 1994. Nowadays, there are some web-based calculators that can be used to calculate CDFS in clinical practice.^[20,21]^

The 3-year CDFS rates in the included breast cancer patients are shown in Figs. 1 and 2. There was a gradual decrease in overall 3-year CDFS. Subgroup analysis also seemed to show a similar trend with a gap within all subgroups except the HR status subgroup. We found that the 3-year CDFS showed a trend for a gradual increase in the HR-negative group and a gradual decrease in the HR-positive group; the 2 CDFS intersected at year 2. Previous studies have reported that the prognosis for breast cancer, including cancer recurrence, differs according to HR status.^[22–24]^ These results suggest that different surveillance plans may be needed for HR-positive and HR-negative patients.

In this study, we also aimed to identify indicators that can predict recurrence in breast cancer patients. We included factors that are accepted indictors of breast cancer prognosis, such as HR and HER2 status, LVI, Ki-67 labeling index, pT stage, and pN stage. Because of clinically used breast cancer markers such as HR and HER2 status change over time during the course of disease progression, prognostic factors used in this study are all baseline values.^[25]^

In our analysis, the risk factors for breast cancer recurrence differed with time. The significant risk factors were present of LVI, elevated Ki-67, high pT stage, and high pN stage at years 0 and 1; present of LVI and high pN stage at year 2; high pN stage at year 3; positive HR status and high pN stage at year 4; and present of LVI at year 5. At years 0 and 1, positive HR status was the only preventive factor identified and at year 5, elevated Ki-67 labeling index was a preventive factor. There was no prevention factor at years 2 to 4. No single prognostic factor could predict breast cancer recurrence at all times up to year 5. For example, positive HR status was identified as a preventive factor in the early years, but this became a risk factor as time passed. These results also changed according to HR status (data not shown).

From the result of Table 1, type of breast cancer showed a different HR in baseline univariate analysis. Total mastectomy patients had a higher recurrence rate from the baseline compared with breast conserving patients. We think that the preoperative tumor burden affects the decision of surgical method before surgery.^[26]^ Patients with more tumor burden will need to receive total mastectomy more frequently.

Breast cancer is known to be heterogeneous,^[5]^ and we performed 3-year CDFS analysis of the molecular subtype. Similar results were found for the 3-year CDFS stratified by molecular subtype. As expected, the 3-year CDFS rates for HER2-positive and triple-negative patients were similar to the rate for the HR-negative group, and the CDFS rates were similar between the luminal A subtype and luminal B subtype patients and the HR-positive patients. This is why we used HR status instead of the molecular subtype to group patients for cancer surveillance.

Our study included a relatively large sample size of 7587 participants. Therefore, the possibility that outliers were included is low. Although no factor could predict recurrence exactly during the long-term follow-up, there seemed to be a trend in the prognostic factors during the follow-up period. Statistically insufficient power in long-term follow-up may be related to the decrease in sample size with time. As shown in Fig. 1 this may be derived from the low “number at risk” as time passes. Therefore, further studies should include longer follow-up to identify indicators of breast cancer recurrence during long-term follow-up of breast cancer patients.

The ASCO and National Comprehensive Cancer Network have released guidelines for breast cancer surveillance.^[26,27]^ Despite minor differences between the guidelines, both recommend history taking and physical examinations every 3 to 12 months for 5 years and then annually. Mammographic evaluation should be performed annually. The use of laboratory or imaging studies for metastases screening, such as complete blood counts, chemistry panels, bone scans, chest radiographs, liver ultrasounds, pelvic ultrasounds, computed tomography scans, positron emission tomography scans, magnetic resonance imaging, and tumor markers is not recommended for routine follow-up in the absence of clinical signs and symptoms that suggest disease recurrence.

Breast cancer-related costs are increasing and this increase is becoming a social burden.^[28–30]^ Most patients experience a fear of recurrence,^[31]^ which is why individual tailored follow-up plans are needed instead of ready-made plans. We aimed to identify whether some patients might need continuous close surveillance whereas, in other patients, it might be possible to omit some tests or extend the intervals between surveillance tests. The results of our study lead us to suggest that minor changes in the guidelines for breast cancer surveillance and the follow-up schedule might be worthwhile.

From the results from our study, we suggest that the surveillance and follow-up plans should be different for HR-positive and HR-negative patients. HR-positive patients need continuous care. Some laboratory or imaging studies to screen for metastases may be included if they have been disease free for >2 years. On the other hand, HR-negative patients may need to have only an annual history and physical examination plan with the same mammography schedule if they have been disease free for >3 years.

In conclusion, in HR-positive patients who are disease free after 3 years, we recommend a more tailored management such as continuous care during surveillance and metastases workup, which is not recommended in the current guidelines. Personalized management may also be possible in HR-negative patients by extending the surveillance period, which may reduce the social costs. Further studies with long-term follow-up are needed to confirm our results.
